# Real-world management of patients with simultaneously diagnosed synchronous liver and lung metastatic colorectal cancer – a national cohort study

**DOI:** 10.2340/1651-226X.2026.45045

**Published:** 2026-03-12

**Authors:** Laia Faseh, Petter Fruhling, Helena Taflin, Jenny Lundmark Rystedt, Caroline Williamsson, Oskar Hemmingsson, Bergthor Björnsson, Marco Gerling, Ernesto Sparrelid, Per Sandström, Kristina Hasselgren, Jennie Engstrand

**Affiliations:** a Division of Surgery and Oncology, Department of Clinical Science, Intervention and Technology, Karolinska Institutet, Karolinska University Hospital, Stockholm, Sweden; b Department of Surgical Sciences, Uppsala University, Uppsala, Sweden; c Department of Surgery, Institute of Clinical Sciences, Sahlgrenska Academy at University of Gothenburg, Sahlgrenska University Hospital, Gothenburg, Sweden, Department of Transplantation, Sahlgrenska University Hospital Gothenburg, Region Västra Götaland Sweden, Gothenburg, Sweden; d Department of Clinical Sciences, Surgery, Lund University, Department of Surgery, Skåne University Hospital, Lund, Sweden; e Department of Diagnostics and Intervention, Surgery, Umeå University, Umeå, Sweden; f Wallenberg Centre for Molecular Medicine, Umeå University, Umeå, Sweden; g Department of Surgery in Linköping and Department of Biomedical and Clinical Sciences, Linköping University, Linköping, Sweden

**Keywords:** colorectal neoplasm, liver neoplasms, secondary, lung neoplasm, secondary, multidisciplinary team, neoplasm metastasis, population-based studies, survival analysis

## Abstract

**Background and purpose:**

Real-world data on management and outcomes of patients with simultaneously diagnosed synchronous colorectal liver and lung metastases are limited. This national study evaluated referral patterns, treatment strategies, and survival in a population-based cohort.

**Patient/material and methods:**

This retrospective cohort study used Swedish national registries to identify patients with synchronous liver and lung metastatic colorectal cancer (CRC), defined as metastases detected within 6 months of CRC diagnosis between 2008 and 2016. Medical record review provided additional information on diagnosis confirmation, multidisciplinary team (MDT) referral, metastatic burden, and treatment. Logistic regression identified factors associated with MDT referral and curative treatment, and Cox regression with a time-varying covariate assessed survival.

**Results:**

Among 2703 registry-identified patients, medical records were accessible for 855. After exclusion of extrahepatic, non-pulmonary metastases, 556 remained for analysis. A total of 189 patients (34%) were discussed at a liver MDT conference. Referred patients were younger, had lower metastatic burden, and better performance status than non-referred. Median survival was 24 months (95% CI [confidence interval] 21–28) for referred versus 10 months (95% CI 7–12) for non-referred patients. Curative local treatment of liver and/or lung metastases was performed in 101 patients (18%), and complete metastasectomy in 34 (6%), conferring superior survival compared with liver-only intervention (hazard ratio 0.34, 95% CI 0.18–0.61). The main reason for non-referral was presumed non-resectability.

**Interpretation:**

Referral to an MDT and subsequent local treatment were associated with improved survival, although this may partly reflect favorable patient and tumor characteristics influencing referral and treatment decisions. Patients with adequate physiological reserve should routinely be evaluated in an organ-specific MDT for potential curative treatment.

## Introduction

Surgical resection of synchronous colorectal liver metastases (CRLM) has demonstrated consistent survival benefits [1]. It has therefore been assumed that metastasectomy of both liver and lung metastases would confer similar survival advantages. This notion is supported by the European Society for Medical Oncology (ESMO) guidelines, which advocate for complete metastasectomy when technically feasible, although these guidelines do not define resectability in terms of the number and extent of metastases [2].

The incidence of *simultaneously* diagnosed *synchronous* liver and lung metastases among all patients with colorectal cancer (CRC) is relatively low, estimated to be around 3.1–3.4% in population-based studies [3–5]. Consequently, data on the outcomes of metastasectomy in synchronous, simultaneous liver and lung metastatic CRC are limited [6].

Most available data on the outcomes of synchronous liver and lung metastasectomy originate from retrospective surgical series, with reported 5-year survival rates ranging from 43 to 72% [7–10]. However, these studies typically involve rigorous selection criteria, excluding patients with unresected primary tumors, non-resectable liver or lung metastases, or non-pulmonary extrahepatic disease [7–9]. This selection bias raises concerns about the generalizability of these findings to a broader population-based setting as the reported survival benefits could reflect the selection of healthier patients with less aggressive disease. Indeed, a recent analysis of the Surveillance, Epidemiology, and End Results (SEER) database did not demonstrate a survival benefit associated with resection of both liver and lung metastases, suggesting that the previously reported survival advantages of resection may not translate into a population-based setting [11]. This contrasts with a Swedish nationwide study, which reported a 5-year survival rate of 74% among those who underwent complete metastasectomy, compared to 2.6% among the non-resected patients [4]. However, that study lacked information on metastatic characteristics and reasons for non‑referral to multidisciplinary team (MDT) conferences, preventing assessment of how many non‑resected patients were potential candidates for curative local treatments.

This study is based on the same national registry cohort as the previous Swedish study [4], but extends that work through comprehensive medical record review, enabling characterization of metastatic burden, referral to MDT conferences and reasons for non-referral.

This nationwide, population‑based study aimed to examine referral patterns, treatment decisions, and survival outcomes for simultaneously diagnosed synchronous colorectal liver and lung metastases. It was hypothesized that complete curative-intent local treatment of both metastatic sites would be associated with superior survival compared to single-organ intervention, and that non-referral to specialist MDT evaluation may contribute to underutilization of potentially curative treatment.

## Patients/material and methods

### Study cohort

This retrospective observational study analyzed synchronously detected liver and lung metastatic CRC over an 8-year period between 2008 and 2016, leveraging data from Swedish national registries. The detailed methodology for creating the study cohort has been previously published [4].

The backbone of the study was data sourced from the Swedish Colorectal Cancer Registry (SCRCR), which provides comprehensive information on each CRC case, including the date of diagnosis, extent of preoperative investigations, liver and lung metastasis status, tumor site and stage, surgical interventions, histopathological findings, and postoperative complications, including referral for metastasectomy. This registry exhibits a completeness of 98.8% when compared annually to the Swedish Cancer Registry [12].

To ensure the inclusion of all patients with liver and lung metastases, data from the SCRCR was linked with the National Patient Register, which covers all inpatient and outpatient hospitalizations in Sweden. Patients with liver (International Classification of Diseases [ICD]-10 code C78.7) and lung (ICD-10 code C78.0) metastases diagnosed within 6 months before or after the diagnosis of CRC were extracted.

Data on liver interventions (resections and/or thermal ablations) were obtained from the National Quality Registry for Liver, Bile Duct, and Gallbladder Cancer (SweLiv). Corresponding data on thoracic interventions were extracted from the National Quality Registry on Thoracic Surgery (ThoR), which has a national coverage of 92.5% [13]. To capture curative-intent local treatments not recorded in SweLiv or ThoR, including thermal ablation and stereotactic body radiotherapy (SBRT) for liver and lung metastases, relevant procedural codes were retrieved from the National Patient Register, as previously described [4]. This information was supplemented and verified through detailed reviews of medical records. In this study, metastasectomy was defined as curative-intent local treatments of liver and/or lung metastases, including surgical resection and thermal ablation for liver metastases and the addition of SBRT for lung metastases.

The registries lack information on the reasons for not referring patients for metastasis surgery, as well as data on the number and size of metastases in the non-resected population, which are pseudo-indicators of resectability. Furthermore, there is no information on operability indicators, such as performance status or American Society of Anesthesiologists (ASA) classification, nor on whether non-resected patients received oncological treatment. To address these gaps, all available electronic medical records were reviewed to collect data on: (1) The accuracy of liver and lung metastases diagnosed within 6 months of CRC diagnosis, (2) The presence of extrahepatic, non-pulmonary metastases, (3) Referral to liver and/or thoracic MDT conferences, (4) The number of metastases and the size of the largest liver metastasis, as well as the reasons for not proceeding with a liver intervention, (5) The number and extent of lung metastases, and (6) Palliative treatment modalities, including systemic oncological therapy or best supportive care.

### Referral and treatment practice in Sweden

In Sweden, a country with a population of approximately 10.5 million, liver and lung interventions are centralized to six University Hospitals that provide care for diverse geographical areas with highly varying population densities. Liver and/or thoracic interventions are not performed outside of the six University Hospitals. The national guidelines on the management of CRLM that applied at the beginning of the study period encouraged referrals to liver and thoracic MDTs at the discretion of the treating physician. In the update of 2012, the national guidelines recommended that all CRLM patients undergo a multidisciplinary assessment by experts in liver surgery, colorectal surgery, gastrointestinal oncology and radiology, prior to treatment initiation. Decisions regarding referral for liver or thoracic MDT conferences are made at local CRC MDTs, led by oncologists and colorectal surgeons.

Ethical approval was obtained from the ethics review board, and the study was conducted in accordance with the World Medical Association Declaration of Helsinki. The manuscript was completed in accordance with the Strengthening the Reporting of Observational Studies in Epidemiology (STROBE) guidelines [14].

### Statistics

Patients’ characteristics were summarized with descriptive statistics: proportions for categorical variables; compared using Chi-squared or Fisher’s exact test, and medians for non-normally distributed continuous variables; compared with the Wilcoxon rank-sum test. Factors associated with metastasectomy were analyzed using logistic regression, presented as odds ratios (OR) and 95% confidence intervals (CI). Cox proportional hazard regression models assessed time-to-event outcomes (death) presented with hazard ratios (HR) and 95% CI calculated from the date of diagnosis of liver metastases. The selection of variables for the multivariable regression models were constrained by the registry-based design. Only variables consistently available for both treated and non-treated patients were considered, excluding collinear variables. Missingness was considered unlikely to be random, as more detailed clinical and radiological data were preferentially documented among patients referred for specialist evaluation and approached 50% for some variables. Multivariable analyses were therefore restricted to complete-case data. For categorical variables, overall associations were evaluated using joint Wald tests. Survival probabilities were estimated using standard Kaplan–Meier graphs, and differences between groups were assessed using the log-rank test. Median follow-up time was calculated using the reverse Kaplan–Meier method.

To mitigate immortality bias when comparing survival between liver-only intervention and complete metastasectomy (including lung interventions), a time-varying Cox regression model was used. Each patient’s observation period was split into pre- and post-lung intervention intervals, allowing patients to transition between risk sets at the time of the second procedure (the lung intervention). The survival, including lung intervention as a time-varying exposure, was visually illustrated with the *extended* Kaplan–Meier method [15]. While extended Kaplan–Meier curves share similar visual characteristics with standard Kaplan–Meier, they cannot generally be interpreted as proper survival functions [16]. Therefore, the extended Kaplan–Meier curves presented should be interpreted as a descriptive tool to visualize the association between surgical intervention and survival, accounting for the time-varying nature of the exposure. All statistical analyses were performed using STATA/MP 18.0, and *p*-values < 0.05 were considered significant.

## Results

### Review of medical records

**Figure 1:**
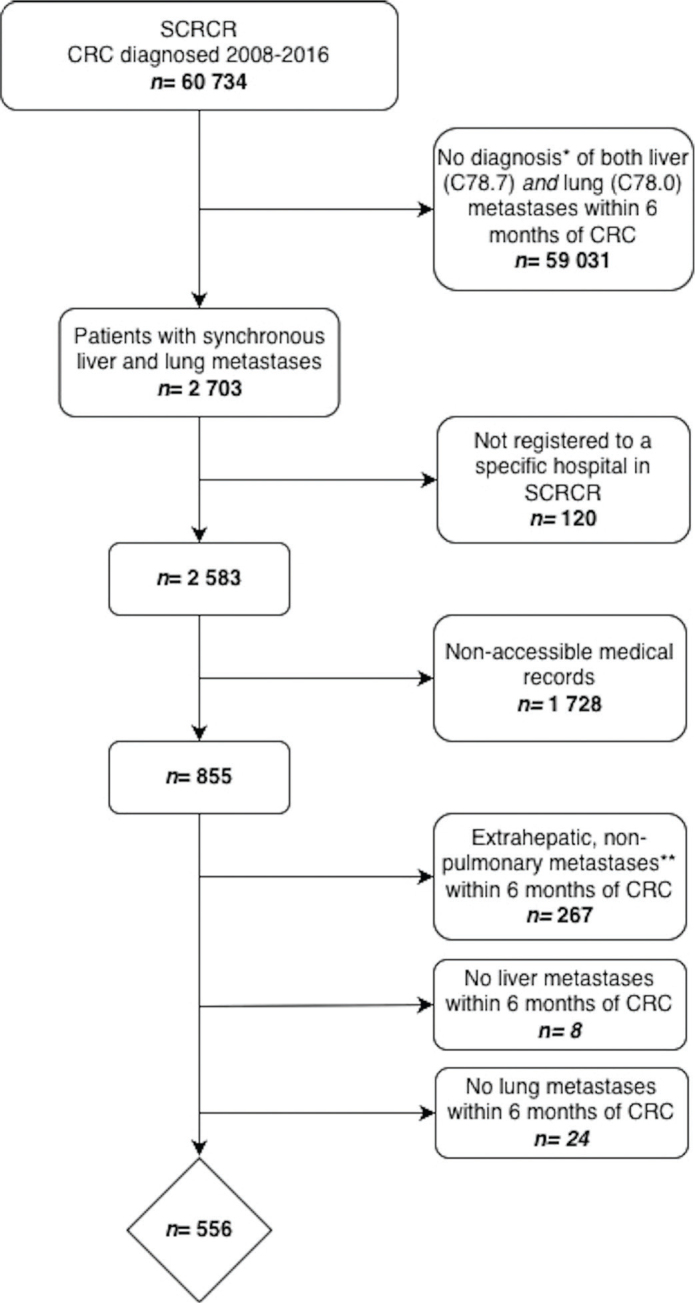
Flow-chart illustrating the identification of patients with synchronous colorectal liver and lung metastases from the Swedish Colorectal Cancer Registry (SCRCR), and subsequent exclusions leading to the final study cohort. Of the 60,734 patients with colorectal cancer (CRC), 2703 were identified with synchronous liver and lung metastases. Patients not registered to a specific hospital (*n* = 120) or with non-accessible medical records (*n* = 1728) were excluded. Among the remaining 855 patients with accessible medical records, those with extrahepatic, non-pulmonary metastases (*n* = 267), no confirmed liver metastases (*n* = 8), or no confirmed lung metastases (*n* = 24) were further excluded, resulting in a final study cohort of 556 patients. *Diagnosis based on ICD codes. **Diagnosis based on review of medical records.

By merging data from the national registries, 2703 patients with synchronous liver and lung metastases were identified across the six healthcare regions (A–F). Of these, 120 patients were excluded as they were not registered to a specific hospital in SCRCR, which precluded assignment to a regional investigator for medical record review (Figure 1). Among the remaining 2583 patients, medical records were accessible for 855 patients (32%), with substantial variation in availability across the healthcare regions, ranging from 18 to 63% (Supplementary Figure 1).

Following exclusion of patients with extrahepatic, non-pulmonary metastases (*n* = 267) and those in whom liver (*n* = 8) and lung (*n* = 24) metastases could not be confirmed within 6 months from CRC diagnosis, 556 patients remained for the final analysis (Figure 1). The composition of the final cohort varied across regions, with Region A contributing 223 patients (40%) and Region D contributing 40 patients (7%) (Supplementary Figure 1). The observed regional differences in patient representation likely reflect variations in accessibility to electronic medical record systems and population size across healthcare regions.

### Patient and tumor characteristics and referral to liver MDT

Of the 556 patients with synchronous liver and lung metastases, 189 patients (34%) had been referred to a liver MDT conference (Table 1). Liver MDT conference referral was significantly associated with younger age, lower ASA scores, better ECOG performance status, fewer and smaller liver metastases, fewer lung metastases, a lower incidence of bilateral lung metastases and higher rates of primary tumor resection (Table 1). All patients who underwent a liver intervention had been discussed in a liver MDT conference prior to surgery. A significantly higher proportion of patients who were discussed at the liver MDT conference also underwent lung metastasectomy (Table 1). The significant regional differences observed in referral rate should be interpreted in the context of the preceding exclusion process and the varying availability of medical records.

**Table 1:** Patient and tumor characteristics in 556 patients with simultaneous synchronously diagnosed liver and lung metastatic colorectal cancer depending on referral to a liver multidisciplinary team meeting.

Variables	All patients *n* = 556	Liver MDT *n* = 189	No liver MDT *n* = 367	*P^*
**Health care region**				
A	223 (40)	43 (23)	180 (49)	< 0.001
B	89 (16)	43 (23)	46 (13)	
C	81 (15)	55 (29)	26 (7)	
D	40 (7)	9 (4)	31 (9)	
E	73 (13)	19 (10)	54 (15)	
F	50 (9)	20 (11)	30 (17)	
**Age, years, median (IQR)**	69 (62, 77)	66 (60, 71)	71 (63, 79)	< 0.001
Missing, *n* (%)	0	0	0	
**Gender, female (% of known)**	235 (44)	81 (44)	154 (44)	0.972
Missing, *n* (%)^^	17 (3)	3 (2)	14 (4)	
**ASA, *n* (% of known)**				
1	69 (20)	33 (24)	36 (17)	0.010
2	138 (39)	60 (44)	78 (36)	
3	130 (37)	43 (31)	87 (41)	
4	14 (4)	1 (1)	13 (6)	
Missing, *n* (%)	205 (37)	52 (28)	153 (42)	
**ECOG, *n* (% of known)**				
0	150 (35)	68 (45)	82 (29)	< 0.001
1	172 (40)	61 (40)	111 (40)	
2	71 (16)	20 (13)	51 (18)	
3–4	38 (9)	3 (2)	35 (13)	
Missing, *n* (%)	125 (22)	37 (20)	88 (24)	
**Chemotherapy, *n* (% of known)**				
Yes	317 (72)	120 (94)	197 (63)	< 0.001
No	122 (28)	7 (5)	115 (37)	
Missing, *n* (%)	117 (21)	62 (33)	55 (15)	
**Primary tumor**				
**Primary tumor location, *n* (% of known)**				
Right colon	127 (23)	41 (22)	86 (23)	0.331
Left colon	177 (32)	68 (36)	109 (30)	
Rectum	251 (45)	80 (42)	171 (47)	
Missing, *n* (%)	1 (0.2)	0	1 (0.3)	
**Treatment of primary tumor, *n* (%)**				
No intervention	264 (47)	72 (38)	192 (52)	< 0.001
Stent	85 (15)	15 (8)	70 (19)	
Deviating stoma	32 (6)	5 (3)	27 (7)	
Resection	175 (31)	97 (51)	78 (21)	
Missing, *n* (%)	0	0	0	
**Liver metastases**				
**No. of liver metastases, *n* (% of known)**				
1	46 (10)	26 (15)	20 (7)	< 0.001
2–5	128 (28)	65 (38)	63 (22)	
6–10	98 (21)	29 (17)	69 (24)	
> 11	187 (41)	49 (29)	138 (48)	
Missing, *n* (%)	97 (17)	20 (11)	77 (21)	
**Size of largest metastasis, mm, median (IQR)**	35 (20, 65)	30 (17, 50)	43 (24, 70)	< 0.001
Missing, *n* (%)	225 (40)	38 (20)	187 (51)	
**Liver intervention**	97 (17)	97 (51)	0	< 0.001
**Lung metastases**				
**Lung MDT**	83 (15)	76 (40)	7 (2)	< 0.001
**Liver metastases**				
**No. of liver metastases, *n* (% of known)**				
1	46 (10)	26 (15)	20 (7)	< 0.001
2–5	128 (28)	65 (38)	63 (22)	
6–10	98 (21)	29 (17)	69 (24)	
> 11	187 (41)	49 (29)	138 (48)	
Missing, *n* (%)	97 (17)	20 (11)	77 (21)	
**Size of largest metastasis, mm, median (IQR)**	35 (20, 65)	30 (17, 50)	43 (24, 70)	< 0.001
Missing, *n* (%)	225 (40)	38 (20)	187 (51)	
**Liver intervention**	97 (17)	97 (51)	0	< 0.001
**Lung metastases**				
**Lung MDT**	83 (15)	76 (40)	7 (2)	< 0.001
**No. of lung metastases, *n* (% of known)**				< 0.001
1	95 (24)	53 (41)	42 (16)	
2–5	170 (43)	58 (45)	112 (43)	
6–10	94 (24)	12 (9)	82 (31)	
> 11	32 (8)	6 (5)	26 (10)	
Missing, *n* (%)	165 (30)	60 (32)	105 (29)	
**Bilateral lung metastases, *n* (% of known)**	276 (67)	67 (50)	209 (75)	< 0.001
Missing, *n* (%)	143 (26)	54 (29)	89 (24)	
**Lung intervention**	38 (7)	35 (19)	3 (1)	< 0.001

MDT: Multidisciplinary Team; ASA: American Society of Anesthesiologists; ECOG: Eastern Cooperative Oncology Group; IQR: interquartile range.

Numbers within brackets are percentages unless otherwise indicated. Liver interventions include surgical resection and thermal ablation. Lung interventions included surgical resection, thermal ablation and stereotactic body radiotherapy.

^ Comparison between patients referred to liver MDT and not.

^^ Sex was missing in 17 cases due to temporary personal identity numbers not encoding sex.

### Metastasectomy

A total of 101 (18%) patients underwent metastasectomy of liver and/or lung metastases, defined as curative intent surgical resection, thermal ablation and SBRT. Among the 38 patients who underwent curative-intent local treatment of lung metastases (Table 1), surgical resection was the predominant modality while one patient was treated with thermal ablation, two with SBRT and one with a combination of resection and SBRT. Specifically, 34 patients underwent complete metastasectomy of both liver and lung metastases, while 63 patients underwent liver-only intervention, and four patients underwent lung metastasectomy only.

Factors associated with metastasectomy (*n* = 101) are detailed in Table 2. In multivariable analysis based on complete-case data (*n* = 244), worse ECOG performance status, a higher number of liver metastases, larger liver metastasis size and bilateral lung metastases were independently associated with a decreased likelihood of undergoing metastasectomy, including local treatments such as thermal ablation and SBRT (Table 2).

**Table 2:** Patient and tumour factors associated with metastasectomy in patients with liver and lung metastatic colorectal cancer diagnosed between 2008 and 2016.

Variables	Univariable analysis			Multivariable analysis**		
	OR	95% CI	*p*	OR	95% CI	*p*
**Age**						
< 50	Ref		0.012	Ref		0.147
51–60	1.08	0.45–2.60		1.42	0.30–6.77	
61–70	0.94	0.43–2.06		2.70	0.63–11.5	
71–80	0.61	0.27–1.40		1.16	0.26–5.21	
> 80	0.04	0.01–0.33		0.28	0.02–3.34	
Male gender	1.14	0.73–1.77	0.562	0.85	0.38–1.90	0.690
ECOG ≥ 1	0.32	0.19–0.53	< 0.001	0.31	0.14–0.70	0.005
Primary tumor location						
Right colon	Ref		0.022	Ref		0.016
Left colon	1.77	0.98–3.18		2.10	0.69–6.40	
Rectum	0.93	0.51–1.67		0.57	0.19–1.68	
**Liver metastases**						
No. of liver metastases						
1	Ref		< 0.001	Ref		< 0.001
2–5	0.46	0.23–0.92		0.45	0.16–1.28	
6–10	0.14	0.06–0.32		0.03	0.01–0.18	
> 11	0.05	0.02–0.11		0.10	0.03–0.34	
Size of largest metastasis > 5 cm	0.32	0.18–0.58	0.001	0.37	0.14–0.96	0.042
**Lung metastases**						
No. of lung metastases*						
1	Ref		< 0.001			
2–5	0.30	0.16–0.53				
6–10	0.07	0.02–0.21				
> 11	0.05	0.01–0.40				
Bilateral lung metastases	0.18	0.10–0.31	< 0.001	0.21	0.09–0.45	< 0.001

OR: Odds ratio; ECOG: Eastern Cooperative Oncology Group Performance Status Scale; CI: confidence interval.

Metastasectomy includes surgical resection, thermal ablation, and stereotactic body radiotherapy.

* Multicollinearity; bilateral location of lung metastases is dependent on no of lung metastases.

** Multivariable model based on 244 complete cases.

### Survival in the entire cohort

**Figure 2:**
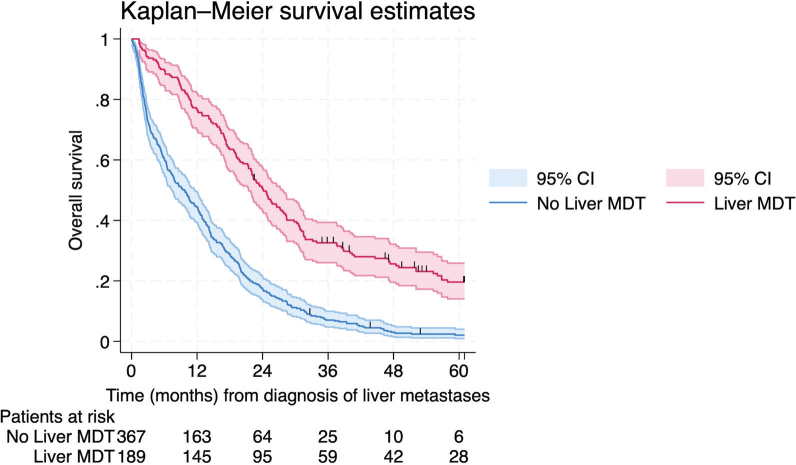
Kaplan–Meier survival estimates of 556 patients depending on referral status to the liver multidisciplinary team meeting, demonstrating a 5-year overall survival of 19.6% (95% CI [confidence interval]: 14.1–25.9%) and 2.1% (95% CI: 1.0–4.0%), respectively.

The median follow-up time, estimated using the reverse Kaplan–Meier method, was 92 months (95% CI 71–128 months). During follow-up, 519 patients died and 37 were alive at end of follow-up. Table 3 presents patient and tumor characteristics, known at diagnosis, associated with overall survival. In multivariable analysis based on 244 complete cases, increasing age, worse ECOG performance status, higher number of liver metastases, and metastasis size >5 cm were independently associated with decreased overall survival (Table 3). Referral to a liver MDT remained associated with improved survival, with a median survival of 24 months (95% CI 21–28 months) compared to 10 months (95% CI: 7–12 months) in those not referred to a liver MDT, *p* < 0.001 (Figure 2). Bilateral lung metastases were not independently associated with survival after adjustment.

**Table 3:** Patient and tumour factors associated with survival in patients with liver and lung metastatic colorectal cancer diagnosed between 2008 and 2016.

Variables	Univariable analysis			Multivariable analysis**		
	HR	95% CI	*p*	HR	95% CI	*p*
Age	1.04	1.03–1.05	< 0.001	1.02	1.01–1.04	0.001
Male gender	0.94	0.79–1.12	0.465	0.86	0.64–1.15	0.303
ECOG ≥ 1	2.16	1.74–2.67	< 0.001	1.79	1.33–2.41	< 0.001
Primary tumor location						
Right colon	Ref		0.023	Ref		0.111
Left colon	0.73	0.58–0.93		0.94	0.64–1.40	
Rectum	0.91	0.73–1.13		1.30	0.91–1.85	
**Liver metastases**						
Liver MDT	0.39	0.32–0.48	< 0.001	0.60	0.44–0.83	0.002
No. of liver metastases						
1	Ref		< 0.001	Ref		< 0.001
2–5	1.50	1.03–2.20		1.56	0.98–2.47	
6–10	2.36	1.59–3.50		1.91	1.14–3.22	
> 11	3.57	2.46–5.16		2.96	1.84–4.76	
Size of largest metastasis > 5 cm	1.65	1.30–2.09	< 0.001	1.61	1.19–2.18	< 0.001
**Lung metastases**						
No. of lung metastases*						
1	Ref		< 0.001			
2–5	1.68	1.28–2.22				
6–10	2.17	1.59–2.95				
> 11	2.25	1.48–3.42				
Bilateral lung metastases	1.77	1.42–2.21	< 0.001	1.16	0.85–1.60	0.347

ECOG: Eastern Cooperative Oncology Group Performance Status Scale; MDT: multidisciplinary team; HR: hazard ratio; CI: confidence interval.

* Multicollinearity; bilateral location of lung metastases is dependent on no of lung metastases.

** Multivariable model based on 244 complete cases.

### Survival in patients undergoing metastasectomy

To explore the potential survival benefit of lung intervention following liver intervention, we included the 95 patients who underwent initial liver intervention. Among these patients, 32 (34%) underwent a subsequent lung intervention after a median interval of 6 months (IQR [interquartile range] 2–12 months). Reasons for not proceeding with the lung intervention could not be further determined from available data. The proportion of patients undergoing complete metastasectomy ranged from 25 to 67% across health care regions (*p* = 0.724).

**Figure 3:**
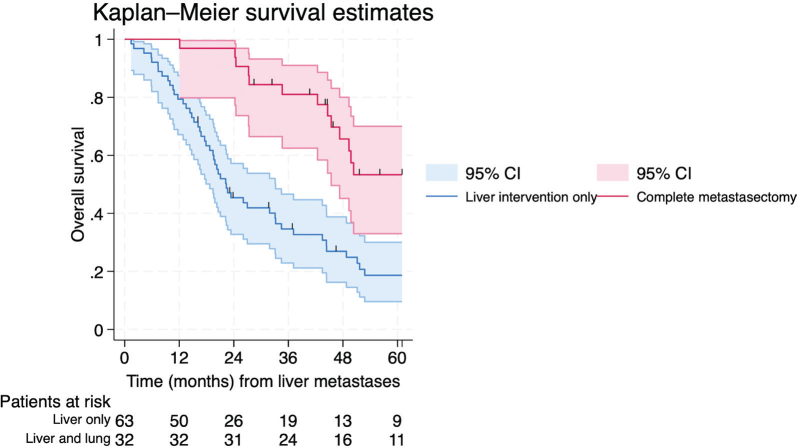
Standard Kaplan–Meier survival estimates of 95 patients categorized at start of follow-up (date of liver intervention) according to future event (subsequent lung intervention). Patients were classified as exposed (future lung intervention) or unexposed (no future lung intervention). This approach introduces immortal time bias, as patients undergoing lung intervention must survive from liver intervention to the time of lung surgery to be classified as exposed. The figure therefore illustrates the bias that can arise when post-baseline events are treated as baseline exposures. The estimated 5-year overall survival was 18.6 (95% CI [confidence interval]: 9.6–30.0%) among patients undergoing liver-only intervention and 53.3% (95% CI: 9.7–70.0%) among those undergoing complete metastasectomy (log-rank test *p* < 0.001).

**Figure 4:**
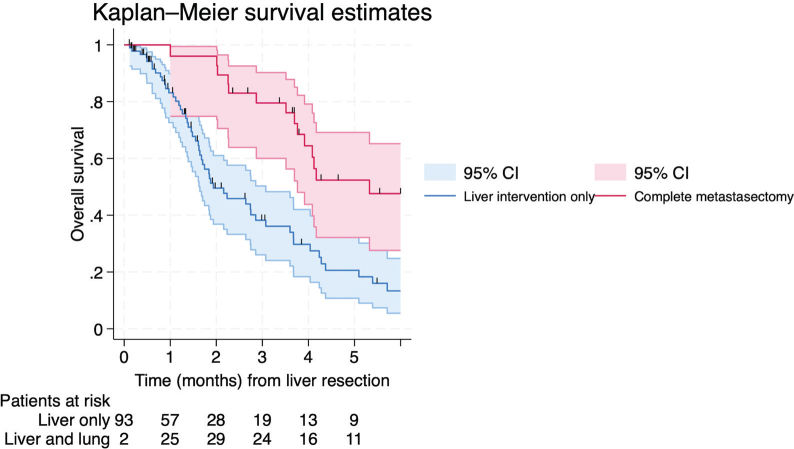
Extended Kaplan–Meier survival estimates of the same 95 patients incorporating lung intervention as a time-varying exposure. In this approach, patient contribute person-time to the unexposed group until the time of lung intervention and thereafter to the exposed group, thereby reducing immortal time bias. Because patients move between exposure states over time, this extended Kaplan-Meier curve does not represent conventional survival functions and should not be interpreted as standard Kaplan-Meier estimated. Differences in patients at risk at specific time-points compared with Figure 3 reflect this dynamic reassignment across risk sets.

When estimating survival from date of liver intervention and categorizing the patients on future events (subsequent lung intervention), patients having complete metastasectomy had a median survival of 64.3 months (95% CI: 45.4-not reached) compared to 22.3 months (95% CI: 18.7–33.2 months) in patients not proceeding with the initially intended lung intervention (log-rank test *p* < 0.001, Figure 3). In the multivariable model restricted to 70 complete cases and limited to ECOG performance status and number of liver metastases due to sample size constrains, lung intervention as a time-varying covariate remained independently associated with improved survival (HR 0.32, 95% CI: 0.16–0.67) (Table 4) as illustrated in an extended Kaplan–Meier curve (Figure 4, for figure interpretation see Figure legend and statistical methods).

**Table 4:** Factors associated with survival in 95 patients diagnosed with liver and lung metastatic colorectal cancer 2008–2016 and undergoing metastasectomy.

Variables	Univariable analysis, standard			Multivariable analysis, standard**		
	HR	95% CI	*p*	HR	95% CI	*p*
Age	1.02	0.99–1.05	0.199			
Male gender	1.03	0.63–1.68	0.909			
ECOG ≥ 1	2.18	1.22–3.90	0.009	2.25	1.23–4.10	0.008
Primary tumor location						
Right colon	Ref		0.523			
Left colon	0.97	0.49–1.94				
Rectum	1.31	0.64–2.66				
**Liver metastases**						
No. of liver metastases						
1	Ref		0.001	Ref		0.400
2–5	1.28	0.67–2.46		1.29	0.65–2.58	
6–10	4.97	2.11–11.68		2.55	0.86–7.57	
> 11	1.11	0.36–3.41		1.57	0.49–5.00	
Size of largest metastasis > 5 cm	1.29	0.68–2.41	0.435			
**Lung metastases**						
No. of lung metastases						
1	Ref		0.596			
2–5	1.56	0.78–3.10				
6–10	1.71	0.50–5.85				
> 11	1.10	0.15–8.29				
Bilateral lung metastases	1.32	0.69–2.50	0.398			
Lung intervention	0.28	0.16–0.52	<0.001	0.27	0.14–0.56	< 0.001
*Lung intervention (as time-varying covariate)* *	*0.34*	*0.18–0.61*	*<0.001*	*0.32*	*0.16–0.67*	*0.002*

ECOG: Eastern Cooperative Oncology Group Performance Status Scale; HR: hazard ratio; CI: confidence interval.

Metastasectomy includes surgical resection, thermal ablation, and stereotactic body radiotherapy.

* In the multivariable model, adjusting for; no of liver metastases and ECOG, HR for lung intervention (as a time-varying covariate) was 0.34 (0.16–0.71), p = 0.004.

** Multivariable model based on 70 complete cases.

### Treatment and survival in the palliative cohort

Palliatively treated patients (*n* = 455) had a median survival of 10.6 months (95% CI: 9.1–12.2 months), estimated from diagnosis of liver metastases. The most common reason for non-referral to a liver MDT (*n* = 367) was the determination of non-resectable disease as assessed by the colorectal surgeon or oncologist (*n* = 319, 87%). Additional reasons for non-referral included severe comorbidity/impaired general condition (*n* = 45, 12%), complete response after neoadjuvant chemotherapy (*n* = 1), patient refusal (*n* = 1), and disease progression during downsizing chemotherapy (*n* = 1). Among the non-referred, 197 patients (54%) were treated with palliative chemotherapy. Data as to why the remaining 170 patients (46%) did not receive palliative chemotherapy could be extracted from medical records of 111 patients (65%). The most frequent cited reasons were impaired general condition/comorbidity (*n* = 78, 70%), followed by advanced age (*n* = 18, 16%) and patient refusal (*n* = 8, 7%). Other reasons were lethal complications following primary tumor resection/stoma creation (*n* = 6, 5%) and asymptomatic patient (*n* = 1, 1%).

## Discussion and conclusion

This national cohort study evaluated MDT conference referral and the associated survival in patients with synchronous colorectal liver and lung metastases, with emphasis on the non-referred/non-resected patients. Approximately one-third of all patients were referred to a liver MDT. As MDT discussion was a prerequisite for surgical intervention of metastases, none of the patients outside the MDT underwent a curative complete metastasectomy. Patients who achieved complete metastasectomy had superior survival compared to those who underwent intervention of one metastatic site or those receiving palliative care treatment. Most non-referred patients were deemed non-treatable in both the liver and lungs by the treating colorectal surgeon or oncologist. Notably, half of the non-referred patients did not receive palliative chemotherapy. Among patients with documented reasons for non-treatment in the present study, impaired general condition or comorbidity was the predominant factor, suggesting that similar considerations may also have applied to patients with missing data. This is in line with population-based data, including a French study in which one third of patients with synchronous metastatic CRC received no chemotherapy and more than half of patients aged ≥75 years did not receive any antitumor treatment [17].

One third of the patients were referred to a liver MDT conference for evaluation, and even fewer were assessed in a thoracic MDT conference. As anticipated, known patient and metastatic characteristics associated with curative-intent interventions and survival correlated with referral to a liver MDT. MDT referral was strongly associated with improved survival outcomes, possibly partly explained by favorable patient and metastases characteristics. The significant regional variability in referral rate must be interpreted in the context of the varying availability of medical records; however, geographic proximity to an hepatobiliary unit likely also contributes to these differences [18, 19]. Since referral to a liver MDT is a prerequisite for curative-intent interventions, all patients fit for liver and lung interventions should be referred for proper evaluation, irrespective of metastatic burden and extent.

International guidelines recommend complete metastasectomy when technically feasible, but do not specify what constitutes resectability by disease extent [2]. Swedish guidelines advice assessing patients with ‘not overly widespread’ metastases in consultation with organ specialists, but this vague term allows for varying interpretations depending on local MDT judgment.

Repeated centralized MDT assessments have been shown to increase resection rates even in oligometastatic disease [20]. In this study, 87% of patients not referred to the liver MDT conference were deemed unresectable by their colorectal surgeon or oncologist. This aligns with previous studies that have demonstrated that a meaningful number of patients with liver metastases are not managed according to best available evidence, and it highlights the importance of evaluation in the setting of a hepatobiliary MDT [21–26]. This has become increasingly important in the light of ongoing development of parenchyma-sparing surgical techniques [27], including local therapies for liver [28, 29] and lung metastases [30, 31], as well as the introduction of liver transplantation [32], although lung metastases currently serve as an exclusion criterion.

Most data on synchronous liver and lung metastasectomy derive from retrospective surgical series, reporting 5-year survival of 43–72% [7–10]. In comparison, this study observed a 5-year survival rate of 53% among the 34 patients undergoing complete metastasectomy. The reported survival gains in surgical cohorts, including this study, likely reflect selection of highly selected patients with favorable tumor biology. A SEER database analysis attempted to clarify the true benefit by matching resected and non-resected patients but was limited by the lack of metastasis characteristics, patient comorbidity and, importantly, resectability status. They found a non-superior median survival of only 1.7 versus 1.5 years. Such unexpectedly low survival among the resected raises questions regarding the curative intent of the metastasectomy, an aspect for which data are missing to properly evaluate [11].

Sweden favors a sequential resection approach for synchronous metastatic CRC, with the liver-first approach currently being the dominant strategy [33, 34]. After primary tumor resection and no evidence of disease progression, the lung intervention is performed. Patients not undergoing the intended lung intervention likely experienced disease progression or complications; however, this remains speculative as comprehensive information was not obtained for a sufficient number of patients. Given that the patients who had liver-only interventions in this study likely experienced disease progression, we could not motivate a matching analysis, comparable to the various matching strategies used in the SEER analysis [11]. Especially as the variables used for matching, known at diagnosis, are likely not the same at time of the second procedure (most often the lung intervention).

To address immortality bias among patients undergoing metastasectomy, patients were split into ‘before’ and ‘after’ exposure groups. Patients contributed to the non-lung treated risk set until the time of their lung intervention, after which they transitioned to the lung-treated risk set, allowing movement across risk sets over time, thereby reflecting the dynamic nature of the exposure. Unfortunately, the extended Kaplan–Meier graphs are less visually intuitive than standard Kaplan–Meier curves [15, 16].

The main limitation of this study was the substantial variability in the accessibility of medical records across different healthcare regions, resulting in a review of only one third of the initial cohort. This variability was largely driven by structural differences in regional electronic medical record systems. In regions with a unified medical record system, it was possible to access records for essentially all citizens, irrespective of whether care was provided at a university or local hospital. In contrast, regions with multiple non-integrated systems limited access to patients managed within the University hospital, from which MDT conferences are conducted. Consequently, patients referred to liver and/or thoracic MDTs were more likely to have accessible medical records, whereas non-referred patients managed exclusively at local hospitals were underrepresented. This introduced potential selection bias, as access to medical records was indirectly associated with referral status and proximity to a University hospital with a hepatobiliary unit, a factor known to influence curative treatment rates and survival outcomes [19]. As a result, the healthcare region could not be reliably evaluated as an independent explanatory variable and was therefore not included in the analyses. In addition, the multivariable analyses were constrained by the registry-based design, as only variables consistently available for both resected and non-resected patients could be included. Because the analyses were restricted to complete cases, resulting in a reduced sample size, the findings should be interpreted as exploratory. Limited data completeness may have introduced residual confounding and reduced statistical precision.

Although a time-varying covariate approach was used to account for immortal time bias, residual and time-varying confounding related to treatment selection cannot be excluded. The decision to perform lung intervention is likely influenced by evolving patient condition and tumor biology, which also affect survival, and the observed associations should therefore not be interpreted as causal. The proportion of missing data ranged from 0 to 51% across variables, and missingness was considered unlikely to be random, as more detailed clinical and radiological information was preferentially available among patients referred for specialist evaluation. Furthermore, several key variables relevant to curative treatment decisions, such as number and size of metastases, ASA classification, and ECOG performance status, are dynamic and may change over time, reflecting evolving clinical assessment and disease progression rather than fixed baseline characteristics. Given these considerations, multiple imputation was deemed inappropriate, and multi-variable analyses were therefore restricted to complete-case data and variables with acceptable completeness. Excluding patients with extrahepatic, non-pulmonary metastases could also be questioned, as surgical treatment of multiple metastatic sites beyond liver and lungs are not contraindicated, although such procedures are reserved for highly selected patients [35]. A further limitation is the restriction of the cohort to patients with only simultaneous synchronous liver and lung metastases, not including metachronous detection. This was a deliberate choice because synchronous disease represents a particularly complex clinical scenario involving indications for systemic chemotherapy, management of the primary tumor and multiorgan resections. The study cohort represents patients diagnosed during 2008–2016. Although surgical treatment strategies for metastatic CRC have evolved since then, the findings remain clinically relevant. Non-referral for MDT evaluation continues to be observed in contemporary practice, underscoring the persistent need for systematic referral of all patients with a potentially treatable disease. The interval between completion of the study cohort and manuscript preparation reflects the complexity of large-scale registry data acquisition, linkage, and validation, as well as the subsequent comprehensive medical record review required to capture key clinical variables not available in national registries. While this inevitably results in a time lag between diagnosis and publication, it enabled a more detailed and clinically informative analysis than would have been possible using registry data alone. An important limitation of this study is also the inability to retrospectively evaluate resectability in non-referred patients within a standardized MDT setting. Incomplete clinical records, variable access to radiological data, and missing information on key treatment decisions precluded reliable assessment of whether MDT referral might have influenced patient outcomes. This is, to our knowledge, the first, population-based study that provides real world data on a national level of multidisciplinary referral behaviors for liver and lung metastatic CRC. The follow-up period further allows for robust assessment of long-term survival.

## Conclusion

This retrospective nationwide study on the management of synchronously diagnosed CRC liver and lung metastases demonstrated that only a small fraction of patients ultimately underwent complete metastasectomy. Whether the observed survival benefit among patients who undergo complete metastasectomy is causally attributable to the surgical interventions remains uncertain, It may instead reflect inherently favorable tumor biology that limits disease progression and thereby permits complete resection. Nevertheless, all patients with reasonable physiological reserve and acceptable functional status to allow for an intervention, should routinely be evaluated in a specialist multidisciplinary setting.

## Supplementary Material



## Data Availability

Following complementary ethical approval (for data sharing) and appropriate DTA, data can be shared.
