# Supplementary material for: Real-world management of patients with simultaneously diagnosed synchronous liver and lung metastatic colorectal cancer – a national cohort study
Source: Acta Oncol. 2026 Mar 12;65:45045. doi: 10.2340/1651-226X.2026.45045 (PMC12993435; doi:10.2340/1651-226X.2026.45045)

Supplementary material has been published as submitted. It has not been copyedited, or typeset by Acta Oncologica

Supplementary Figure 1: Flow-chart illustrating the initial study cohort, the stepwise exclusion process, and the variability in medical record accessibility across the six Swedish health care regions (A-F). The chart details, for each region, the number of patients excluded per region due to non-accessible medical records, and subsequent exclusions related to diagnosis verification, extrahepatic non-pulmonary metastases, or absence of confirmed liver and/or lung metastases.

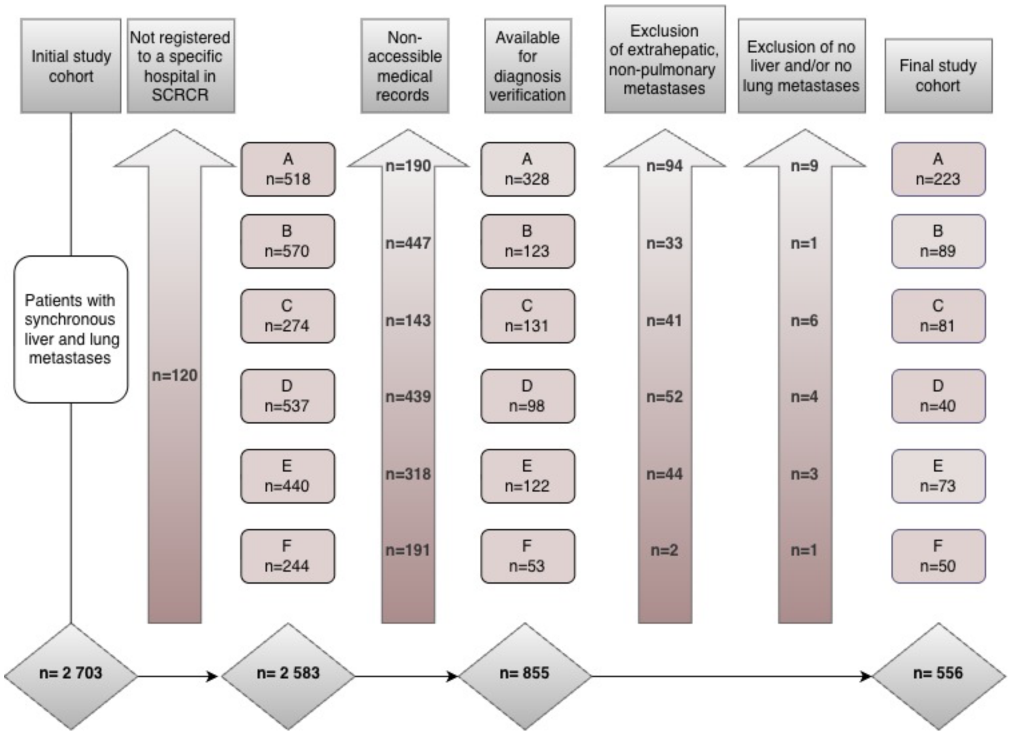

Supplement: Supplementary file 1 [file AO-65-45045-s1.pdf]
